# The Expression and Clinical Outcome of pCHK2-Thr68 and pCDC25C-Ser216 in Breast Cancer

**DOI:** 10.3390/ijms17111803

**Published:** 2016-10-28

**Authors:** Huayong Jiang, Bin Wang, Fuli Zhang, Yuanyu Qian, Chia-Chen Chuang, Mingzhen Ying, Yajie Wang, Li Zuo

**Affiliations:** 1 Department of Radiation Oncology, PLA Army General Hospital, Beijing 100700, China; doctorjhy@163.com (H.J.); radiozfli@163.com (F.Z.); 2 Department of Oncology, Changhai Hospital, The Second Military Medical University, Shanghai 200433, China; qcwangb@163.com (B.W.); yingmz@163.com (M.Y.); 3 Department of Emergency, Chinese PLA General Hospital, Beijing 100853, China; qyy301@sina.com; 4 Radiologic Sciences and Respiratory Therapy Division, School of Health and Rehabilitation Sciences, The Ohio State University College of Medicine, Columbus, OH 43210, USA; chuang.107@osu.edu

**Keywords:** pCHK2-Thr68, pCDC25C-Ser216, immunohistochemistry, breast cancer, genomic instability

## Abstract

Checkpoint kinase 2 (CHK2) and cell division cycle 25C (CDC25C) are two proteins involved in the DNA damage response pathway, playing essential roles in maintaining genome integrity. As one of the major hallmarks of abnormal cellular division, genomic instability occurs in most cancers. In this study, we identified the functional expression of pCHK2-Thr68 and pCDC25C-Ser216 in breast cancer, as well as its association with breast cancer survival. Tissue microarray analysis using immunohistochemistry was constructed to identify the expression of pCHK2-Thr68 and pCDC25C-Ser216 in 292 female breast cancer patients. The relationship among protein expression, clinicopathological factors (e.g., human epidermal growth factor receptor 2 (HER 2), tumor size, tumor-node-metastasis (TNM) classification), and overall survival of the breast cancer tissues were analyzed using Pearson’s χ-square (χ^2^) test, Fisher’s exact test, multivariate logistic regression and Kaplan–Meier survival analysis. Significantly higher expressions of pCHK2-Thr68 and pCDC25C-Ser216 were observed in the nucleus of the breast cancer cells compared to the paracancerous tissue (pCHK2-Thr68, 20.38% vs. 0%; pCDC25C-Ser216, 82.26% vs. 24.24%). The expression of pCHK2-Thr68 and pCDC25C-Ser216 in breast cancer showed a positive linear correlation (*p* = 0.026). High expression of pCHK2-Thr68 was associated with decreased patient survival (*p* = 0.001), but was not an independent prognostic factor. Our results suggest that pCHK2-Thr68 and pCDC25C-Ser216 play important roles in breast cancer and may be potential treatment targets.

## 1. Introduction

As the most common form of malignant cancer, breast cancer is the leading cause of tumor-related deaths in women worldwide. It is estimated that more than 249,260 new cases of breast cancer will be diagnosed in the United States in 2016 and approximately 40,890 estimated deaths from the disease [1]. The diagnosis of breast cancer has increased by 5% annually in developing countries [2]. Therefore, studies on the pathogenesis and development of breast cancer remain ongoing and crucial.

Genomic instability is recognized as a hallmark of malignant tumors, which stem from mutations in the genes related to DNA repair and subsequent cell cycle checkpoint protein malfunctions [3,4]. Timely and accurate DNA repair is necessary to ensure genomic stability; failure in this process can result in adverse effects such as mutation accumulation and neoplastic transformation [5,6]. Accordingly, increasing evidence indicates that abnormal DNA repair is closely related with both onset and progression of breast cancer [7,8].

Among several proteins involved in cell division, the highly regulated cell division cycle 25 (CDC25) phosphatase is regarded as a key component in ensuring normal cell division and genomic integrity [9,10]. CDC25 phosphatase activates cyclin-dependent kinase (CDK)–cyclin complexes and regulates cell cycle progression by driving G1-S/G2-M transitions during mitosis. In response to DNA damage, inhibitory checkpoint kinase (CHK) phosphorylates CDC25 and inhibits its activities, leading to cell cycle arrest. Moreover, the halted cell cycle allows the cell to repair DNA before replication or alternatively undergo apoptosis [9,10]. It is therefore conceivable that dysfunction of these regulatory proteins can result in the accumulation of mutations over time and subsequently contribute to tumorigenesis. In fact, CDC25 overexpression has been implicated in various cancers, such as breast cancer, and is typically associated with poor prognosis [9,11].

Although the upregulated CDC25A and CDC25B isoforms are commonly reported in breast carcinoma, CDC25C is another, less recognized oncogenic isoform [12,13]. Indeed, limited studies have emphasized on the evaluation of CDC25C expression in cancers [14]. It is gradually recognized that the regulation of CDC25C expression in response to DNA damage in cancer cells involves complex processes that are yet to be explored (e.g., alterative splicing) [14]. In addition, CDC25C may be related to the breast cancer due to the potential roles of CDC25C and pCDC25C (Ser216) in certain cancer pathways in women (e.g., vulvar carcinomas) [10,13]. Considering the possible underrated measurement of CDC25C expression in most studies and the prospective involvement of CDC25C in female cancers, we focus our priorities on CDC25C in the carcinogenesis of breast cancer.

The activation of the ATM/ATR–CHK1/2–CDC25C pathway following DNA damage is one of the essential mitotic checkpoint mechanisms [15]. Specifically, CHK2 phosphorylates CDC25C at Ser 216, which subsequently leads to the binding of 14-3-3 proteins and the cytoplasmic sequestration of CDC25C, thereby inhibiting mitotic entry [16]. Previous studies on the relationship between CHK2 and malignant tumors have been primarily focused on gene polymorphisms and mRNA expressions [17,18,19,20,21,22]. However, few studies correlated the phosphorylation of CHK2 and CDC25C with breast cancer. The exact roles of CHK2 and CDC25C in breast cancer have not been fully elucidated. Thus, in this study, we aim to delineate the phosphorylation of CHK2 and CDC25C in breast cancer using immunohistochemistry, as well as expound the role of these proteins in breast cancer development.

## 2. Results

### 2.1. Analysis of Clinical Data

The color reaction/positive staining of pCHK2-Thr68 and pCDC25C-Ser216 were observed in 265 tumor tissue samples and 33 paracancerous tissue samples among the total 292 breast cancer cases studied. Among these 265 tumor tissue samples, 226 (85.3%) were invasive ductal carcinoma (IDC) and 39 (14.7%) were non-IDC. Additionally, 115 (43.4%) of these patients were premenopausal and 150 (56.6%) were postmenopausal patients. Histological grades I, II, and III accounted for 2.3%, 70.5%, and 27.2% of the cases, respectively. TNM stages I, II, and III accounted for 21.5%, 54.7%, and 23.8% of the cases, respectively.

### 2.2. Expression of pCHK2-Thr68 and pCDC25C-Ser216

**Figure 1:**
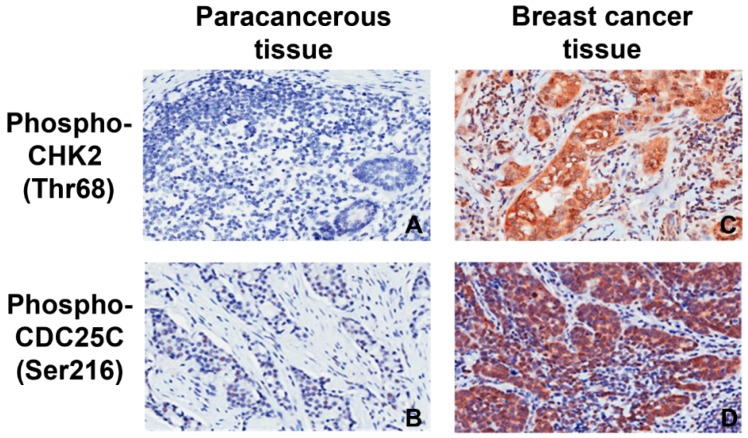
Representative pictures of the immunohistochemical staining of: pCHK2-Thr68 (**A**); and pCDC25C-Ser216 (**B**) in paracancerous tissues. pCHK2-Thr68 (**C**); and pCDC25C-Ser216 (**D**) staining in breast cancer tissues. Original magnification, ×200.

The nuclear staining for pCHK2-Thr68 and pCDC25C-Ser216 was identified in the breast cancer tissues (Figure 1). Table 1 suggests that the expression rates of these two proteins are higher in cancerous tissues than paracancerous tissues (pCHK2-Thr68: 20.4% vs. 0.0%; pCDC25C-Ser216: 82.3% vs. 24.2%, *p* < 0.001). Among 265 cases, higher pCHK2-Thr68 expression was observed in triple-negative breast cancer (TNBC; 15 of 46 total TNBC cases) tissues compared to non-TNBC (39 of 219 total non-TNBC cases) tissues (32.6% vs. 17.8%, χ^2^ = 5.13, *p* = 0.023; Table 2). TNBC cases were identified as estrogen receptor (ER), progesterone receptor (PR), and epidermal growth factor receptor 2 (HER2) negative.

### 2.3. pCHK2-Thr68 and pCDC25C-Ser216 in Relation to Clinicopathological Factors

The clinicopathological factors used in the current study include the following: age at diagnosis, tumor size, number of lymph metastases, TNM stage, pathology type, histology grade, HER2, ER, PR, and menopausal status. The values assigned to these variables were as follows: tumor size (≤2 cm, scored as 1; 2–5 cm, scored as 2; >5 cm, scored as 3), axillary lymph node metastasis (0, scored as 1; 1–3, scored as 2; 4–9, scored as 3; ≥10, scored as 4), age at diagnosis (≤40 years, scored as 1; 41–60 years, scored as 2; >60 years, scored as 3), and histological grade (I, scored as 1; II, scored as 2; III, scored as 3). For pCHK2-Thr68, pCDC25C-Ser216, ER, PR, and HER2, low/undetectable or negative expressions were assigned with 1 (visual scoring ≤ 4), while high or positive expressions (visual scoring ≥ 5) were assigned with 2. Table 3 summarizes the association of the studied factors with expression of CHK2-Thr68 and pCDC25C-Ser216 as evaluated by immunostaining methods. No significant difference was observed between clinicopathological factors and protein expression, suggesting that the expression of both pCHK2-Thr68 and pCDC25C-Ser216 is not related to the metastasis of breast cancer. A positive correlation was found between pCHK2-Thr68 and pCDC25C-Ser216 expressions (*p* = 0.026). The results from multivariate analysis confirm that pCHK2-Thr68 is closely related to the expression of pCDC25C-Ser21 (*p* < 0.05, Table 4)*.*

### 2.4. Survival Analysis

**Figure 2:**
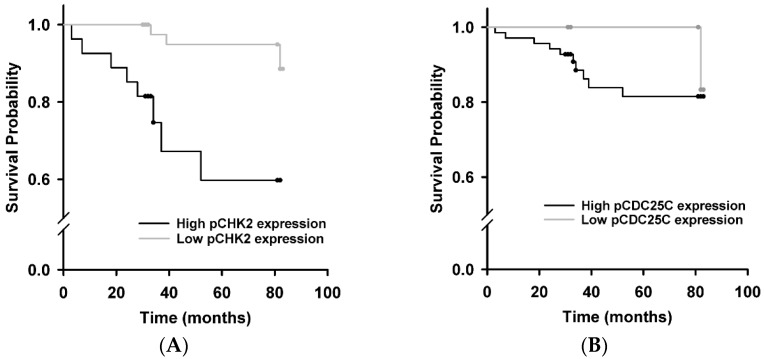
Survival curves using the Kaplan–Meier method: (**A**) Kaplan–Meier curve of overall survival in relation to pCHK2-Thr68 protein expression levels (*p* < 0.001); and (**B**) Kaplan–Meier curve of overall survival in relation to pCDC25C-Ser216 protein expression levels (*p* = 0.392).

Kaplan–Meier survival analysis was performed on 82 cases with follow-ups. The last follow-up was carried out on 31 December 2011. Figure 2A shows a low pCHK2-Thr68 expression observed in patients with a mean survival period of 80.93 months (95% CI: 77.27–83.78). This is longer than the high expression group with a mean survival period of 60.92 months (95% CI: 48.88–72.96), χ^2^ = 11.62, *p* = 0.0001. However, pCDC25C-Ser216 expression was not related to survival (χ^2^ = 0.73, *p* = 0.392; Figure 2B). Cox proportional hazard regression models were implemented to analyze prognostic factors, using entry and exclusion criteria of 0.1 and 0.15, respectively. The results show that pCHK2-Thr68 and pCDC25C-Ser216 expressions are not independent prognostic factors.

## 3. Discussion

**Figure 3:**
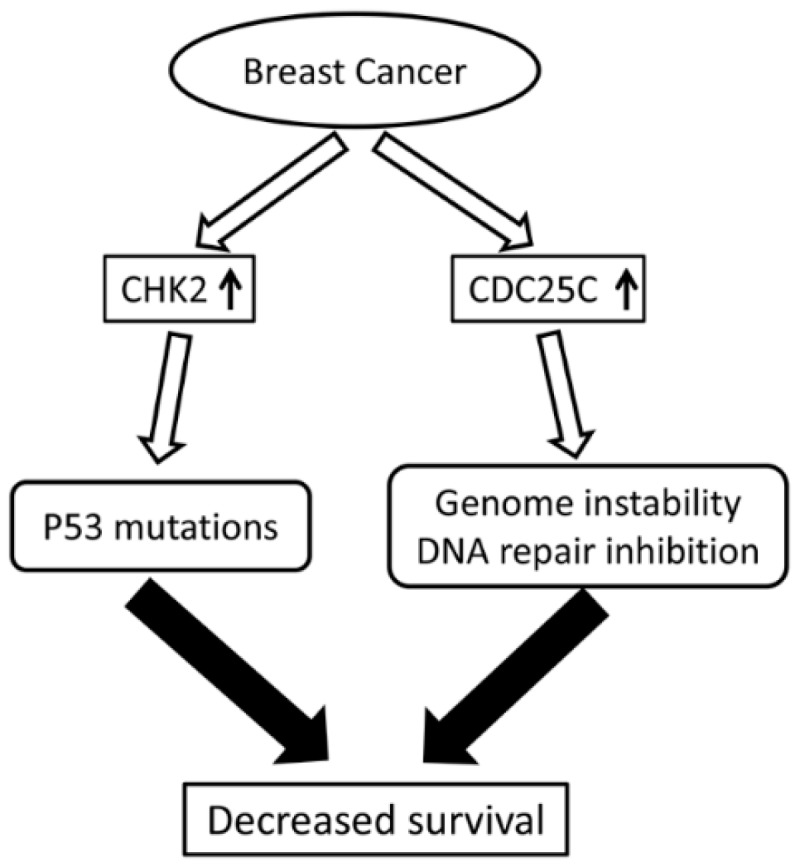
An overview of ATM/ATR-CDK2-CDC25C pathway in addition to the results of the study. Arrows inside the boxes indicate increased levels of CHK2 and CDC25C; all other arrows indicate “leading to”.

The highly conserved CDC25 phosphatase family is known to play an essential role in cell cycle regulation. Notably, dysregulated CDC25 has been implicated in various cancers such as ovarian and vulvar carcinoma due to induced genomic instability [10,13]. In this study, we detected the expression of pCHK2-Thr68 and pCDC25C-Ser216 in both breast cancer and paracancerous tissues using immunohistochemistry and explored their associations with breast cancer metastasis. Our results showed that both pCHK2-Thr68 and pCDC25C-Ser216 expressions were upregulated in cancerous tissues (Figure 3). However, these increased protein expressions are not significantly correlated with the clinicopathological factors of breast cancer.

The activation of DNA damage repair in tumor such as breast cancer serves to restore genetic integrity and impede tumor progression [23]. Therefore, the expression of phosphorylated CHK2/CDC25C is expectedly increased. The elevated levels of pCHK2-Thr68 in the breast cancer tissues in this study are highly consistent with previous results [24]. In addition, we observed a higher expression of pCHK2-Thr68 in TNBC tissues than non-TNBC tissues. Greater activation of the cell cycle checkpoint protein (e.g., CDK) may be associated with the increased activity of TNBC stem cells [25,26] as accumulated *p53* tumor suppressor gene and *BRCA1* gene mutations may trigger ATM/ATR–CHK1/2–CDC25C pathways [27,28]. Such increases in pCHK2-Thr68 also occur in other tumors such as mucinous adenocarcinomas and colorectal cancer [29,30,31].

However, the correlation between clinicopathological factors and pCHK2-Thr68 protein expression may differ in various types of tumors. For example, Bartkova et al. observed higher CHK2 activation in the early TNM stages of bladder cancer [32], while Kshirsagar et al. found elevated expression of pCHK2-Thr68 in high-grade serous ovarian carcinoma [33]. It is likely that persistent activation of CHK2 selectively pressured the cells to mutate a tumor protein *p*53, thereby contributing to tumor development (Figure 3) [34,35]. In the current study, our results distinctly showed that the expression level of pCHK2-Thr68 is not related with clinicopathological factors of breast cancer. Low expressions of pCHK2-Thr68 with higher survival rate may be attributed to the less active cancer cells in certain patients, thereby triggering DNA damage repair mechanism in a smaller extent.

We also did not observe the correlation between pCDC25C-Ser216 expression and clinicopathological factors. It is still unclear as to whether the overexpression of pCDC25C-Ser216 promotes genome instability and tumorigenesis or if it exerts anticancer effects. An overexpression of pCDC25C-Ser216 in patients with later clinical stages of vulvar cancer and lymph node metastases indicates a relation with tumor development [13]. It is likely that pCDC25C-Ser216 remained in the nucleus (without cytoplasmic sequestration) contributes to the cancer development by activating CDK1/cyclin B complex and leading to an un-thorough G2 arrest [13]. In accordance with the study of vulvar carcinoma performed by Wang et al. [13], we observed high expression of pCDC25C-Ser216 with lower survival rate in breast cancer patients. Moreover, our study suggested a positive correlation between the expression of pCHK2-Thr68 and pCDC25C-Ser216 in breast cancer. However, the role of the CHK2/CDC25C pathway in breast cancer requires further elucidation since CDC25C is also closely regulated by other proteins such as checkpoint kinase 1 (CHK1), *p*53, c-Jun N-terminal kinases, and CDK [36]. The results of current study provide further evidence for additional CDC25C activation pathways, with expression rates of 82.3% of pCDC25C-Ser216 and only 20.4% of pCHK2 (see Table 1), further demonstrating the presence of other CDC25C activation pathways.

Chemoradiotherapy plays an important role in the treatment of breast cancer; understanding genomic instability may lead to more effective treatment options. For example, increased expression of CDC25 protein is likely associated with higher radiosensitivity, as it promotes the transition of a cell to a more radiosensitive M phase [37]. Therefore, it is suggested that the activation of CDC25, which inhibits DNA damage repair, can be used as an adjuvant to DNA damaging drug treatment and radiotherapy [9].

In recent years, molecular therapies targeting some of the cell cycle checkpoints have been investigated in phase I or II clinical trials without any significant breakthrough [38]. Intervention targeting the inhibition of abnormal activation of CHK2 may improve pCHK2 expression and prognosis of patients [39]. High expressions of pCHK2 may be accompanied by increased breast cancer susceptibility [40]. Since reactive oxygen species (ROS) play an important role in DNA damage/repair, cell signaling cascades, as well as other key biological activities [41,42], intercorrelations between CDC25/CHK2, antioxidant and ROS need to be thoroughly explored. Although our study found no marked difference between clinicopathological factors and high pCHK2/pCDC25C expression, a positive correlation between these two proteins suggests their possible roles in the DNA repair in breast cancer. Therefore, CHK2 can be a potential therapeutic target for efficient DNA damage repair in patients with breast cancer.

## 4. Materials and Methods

### 4.1. Patients

A total of 292 female breast cancer patients undergoing surgery from January 2001 to June 2011 in Changhai Hospital were enrolled in this study. To test our hypothesis, each tumor was classified according to the American Joint Committee on Cancer (AJCC) tumor-node-metastasis (TNM). The study inclusion criteria were no clear family history of breast cancer and the use of surgery as initial treatment. Patients with stage IV of the disease, a non-curative resection, and those treated with preoperative radiotherapy or chemotherapy, were all excluded. The median patient age was 53 (range 31–84) years. An informed written consent in accordance with Changhai Hospital Human Subjects Review Board was obtained from each subject prior to his or her participation. The study was approved by Second Military University Biomedical Research Ethics Committee and Human Subjects Institutional Review Board of Changhai Hospital (IRB #: 2013310; Approval date: 10 March 2013).

### 4.2. Experimental Methods

#### 4.2.1. Collection of Specimens

Tissue samples were obtained via surgical resection of patients’ breast cancer tumors. Each pathological specimen was divided into approximately four segments, including two breast cancer tissues and two paracancerous tissues (within 2 cm from the edge of the tumor). These tissues were then transferred into a 10% neutral buffer formalin solution. Each segment was sectioned into a dimension of 5 mm × 15 mm × 15 mm. Tissue specimens were dehydrated using the Leica tissue processor (ASP300s, Nussloch, Germany) and embedded with an automated Leica paraffin-embedding machine (Model EG1150, Nussloch, Germany). The pathological paraffin slices were then cut into 4-µm-thick sections and stained with haematoxylin and eosin (HE).

#### 4.2.2. Tissue Chip and Immunohistochemical Staining (IHC)

Tumor cell analysis was conducted via large-core tissue microarray (TMA, OriGene, Rockville, MD, USA). TMA blocks were constructed by extracting 1.5-mm-diameter cylinders from the center of the tumor. A tissue array instrument (Model # ATA-27, Beecher Instruments, Sun Prairie, WI, USA) was used to extract tissue cores and arrange them into blank recipient paraffin blocks. The TMA blocks were cut into 4-µm sections and processed for IHC. Antibodies, including ER antibody (dilution 1:50), PR antibody (dilution 1:50), HER2 antibody (dilution 1:50), pCHK2-Thr68 and pCDC25C-Ser216 antibody (dilution 1:40) were all purchased from Cell Signaling Technology (CST, Boston, MA, USA). Immunostaining was performed using the Envision System with diaminobenzidine (Dako, Glostrup, Denmark).

The protein expression of pCHK2-Thr68 and pCDC25C-Ser216 was determined semi-quantitatively by two senior pathologists who were blinded to the clinicopathological status of the participants. The staining was quantified using visual scoring for the staining intensity (absent, 0; light brown, 1; brown, 2; dark brown, 3) and the extent of staining (percentage of positive tumor cells; ≤10%, 0; 11%–25%, 1; 26%–50%, 2; 51%–75%, 3; >75%, 4). The product of the two scores ranged from 0 to 12. High expression was classified with a score of ≥5 and low expression with a score of ≤4.

#### 4.2.3. Statistical Analysis

Statistical analysis was performed using SPSS18.0 software. Associations between the expression of pCHK2-Thr68 or pCDC25C-Ser216 and the clinicopathological variables were evaluated by either Pearson’s χ-square (χ^2^) test or Fisher’s exact test. Multivariate logistic regression analysis was subsequently conducted to determine the relationship between the expression of pCDC25C-Ser216 and pCHK2-Thr68. The Kaplan–Meier method was used to analyze the overall survival in relation to pCHK2-Thr68 protein expression. The Kaplan–Meier estimates and the Log-Rank tests were used to evaluate the survival data. Cox proportional hazard regression analysis was performed to analyze independent prognostic factors. For all data, *p* < 0.05 were considered statistically significant.

## 5. Conclusions

In this study, clinical specimens were used to confirm the roles of pCHK2-Thr68 and pCDC25C-Ser216 in the development and progression of breast cancer. Elevated pCHK2-Thr68 and pCDC25C-Ser216 expressions were observed in breast cancer tissues compared to paracancerous tissues, which are likely associated with decreased survival in breast cancer patients. However, additional studies are necessary to investigate the underlying mechanisms related to checkpoint pathways and to further elucidate the etiology of breast cancer.

## Figures and Tables

**Table 1:** Expression of pCHK2-Thr68 and pCDC25C-Ser216 in positively staining breast cancer and paracancerous tissues.

	pCHK2-Thr68 Expression		pCDC25C-Ser216 Expression		Total
	High	Low	High	Low	
Breast Cancer	*n* = 54 (20.4%)	*n* = 211 (79.6%)	*n* = 218 (82.3%)	*n* = 47 (17.7%)	*n* = 265
Paracancerous tissues	*n* = 0 (0%)	*n* = 33 (100%)	*n* = 8 (24.2%)	*n* = 25 (75.8%)	*n* = 33
χ^2^/*p* value	8.213/0.004		53.916/0.000		

The current study analyzed 33 normal tissues for phospho-CHK2 expression; while no case of phospho-CHK2 expression was detected among all these 33 samples (Table 1). Therefore, 0 out of 33 indicates a low expression rate for pCHK2 expression in “normal” tissues. High expression of pCHK2-Thr68 has been observed in 54 out of 265 total cases (20.38%) and all cases of paracancerous tissue exhibit low expression, suggesting the activation of CHK2 in the breast cancer cells. Such activation is not shown in normal or paracancerous tissue (0% in high expression of pCHK2-Thr68; Table 1). Therefore, we are referring to the comparison between numbers of the cases with high and low expressions, not to the actual expression intensity ratio between the two. Additionally, in Table 1, we also do not calculate the ratio of the case numbers between cancer and normal tissues nor do we compare the expression signals between the two.

**Table 2:** Expression of pCHK2-Thr68 in TNBC and non-TNBC tissues.

	pCHK2-Thr68 Expression		Total
	High	Low	
TNBC	*n* = 15 (32.6%)	*n* = 31 (67.4%)	*n* = 46
non-TNBC	*n* = 39 (17.8%)	*n* = 180 (82.2%)	*n* = 219
χ^2^/*p* value	5.13/0.023		

TNBC, triple negative breast cancer.

**Table 3:** Correlation between high/low (referring number of cases) pCHK2-Thr68 and pCDC25C-Ser216 expression and clinicopathological factors in 265 breast cancer tumor tissues.

Characteristics	pCHK2-Thr68 (*n*)				pCDC25C-Ser216 (*n*)			
	High	Low	χ^2^	*p* Value	High	Low	χ^2^	*p* Value
Age at diagnosis (years)								
≤40	4	16	2.637 *	0.282	5	15	1.176 *	0.554
40–60	30	140			28	142		
>60	20	55			14	61		
Tumor size (cm)								
≤2	18	70	3.977	0.137	13	175	1.156 *	0.563
2–5	28	127			31	124		
>5	8	14			3	19		
Number of lymph metastases								
0	29	116	0.534 *	0.923	29	116	6.834 *	0.071
1–3	14	53			7	60		
4–9	7	22			4	25		
≥10	4	20			8	16		
TNM stage								
1	10	47	0.423	0.809	12	45	1.446	0.485
2	30	115			22	123		
3	14	49			13	50		
Pathology type								
IDC	49	177	1.610	0.205	39	187	0.242	0.623
Non-IDC	5	34			8	31		
Histology grade								
I	0	6	1.507 *	0.441	1	5	0.371 *	0.885
II	37	150			32	155		
III	17	55			14	58		
HER2								
+	17	53	1.096	0.316	14	56	0.896	0.344
-	37	158			33	162		
ER								
+	25	128	3.637	0.057	25	128	0.484	0.487
-	29	83			22	90		
PR								
+	27	129	2.203	0.138	24	132	1.437	0.231
-	27	82			23	86		
Menopausal status								
Premenopausal	20	95	1.117	0.291	15	100	3.066	0.08
Postmenopausal	34	116			32	118		

* Fisher’s exact probability; ER, estrogen receptor; HER2, human epidermal growth factor receptor 2; IDC, invasive ductal carcinoma; PR, progesterone receptor. + and −, high/positive and low/negative expression.

**Table 4:** Multivariate logistic regression analysis.

Dependent Variable	Independent Variable	Regression Coefficient	SE	*p* Value	OR	95% CI
pCHK2-Thr68	pCDC25C-Ser216	0.849	0.312	0.006	2.337	1.267–4.309
pCDC25C-Ser216	pCHK2-Thr68	1.085	0.337	0.002	2.524	1.462–4.625

CI, confidence interval; OR, odds ratio; SE, standard error.

## References

[1] Siegel R.L., Miller K.D., Jemal A. (2016). Cancer statistics, 2016. CA Cancer J. Clin..

[2] Coughlin S.S., Ekwueme D.U. (2009). Breast cancer as a global health concern. Cancer Epidemiol..

[3] Negrini S., Gorgoulis V.G., Halazonetis T.D. (2010). Genomic instability—An evolving hallmark of cancer. Nat. Rev. Mol. Cell Biol..

[4] Ferguson L.R., Chen H., Collins A.R., Connell M., Damia G., Dasgupta S., Malhotra M., Meeker A.K., Amedei A., Amin A. (2015). Genomic instability in human cancer: Molecular insights and opportunities for therapeutic attack and prevention through diet and nutrition. Semin. Cancer Biol..

[5] Hoeijmakers J.H. (2001). Genome maintenance mechanisms for preventing cancer. Nature.

[6] Bartek J., Lukas J., Bartkova J. (2007). DNA damage response as an anti-cancer barrier: Damage threshold and the concept of “conditional haploinsufficiency”. Cell Cycle.

[7] Ralhan R., Kaur J., Kreienberg R., Wiesmuller L. (2007). Links between DNA double strand break repair and breast cancer: Accumulating evidence from both familial and nonfamilial cases. Cancer Lett..

[8] Hu J., Wang N., Wang Y.J. (2013). XRCC3 and RAD51 expression are associated with clinical factors in breast cancer. PLoS ONE.

[9] Boutros R., Lobjois V., Ducommun B. (2007). CDC25 phosphatases in cancer cells: Key players? Good targets?. Nat. Rev. Cancer.

[10] Sur S., Agrawal D.K. (2016). Phosphatases and kinases regulating CDC25 activity in the cell cycle: Clinical implications of CDC25 overexpression and potential treatment strategies. Mol. Cell. Biochem..

[11] Kristjansdottir K., Rudolph J. (2004). Cdc25 phosphatases and cancer. Chem. Biol..

[12] Ito Y., Yoshida H., Uruno T., Takamura Y., Miya A., Kuma K., Miyauchi A. (2004). Expression of cdc25A and cdc25B phosphatase in breast carcinoma. Breast Cancer.

[13] Wang Z., Trope C.G., Florenes V.A., Suo Z., Nesland J.M., Holm R. (2010). Overexpression of CDC25B, CDC25C and phospho-CDC25C (Ser216) in vulvar squamous cell carcinomas are associated with malignant features and aggressive cancer phenotypes. BMC Cancer.

[14] Albert H., Battaglia E., Monteiro C., Bagrel D. (2012). Genotoxic stress modulates CDC25C phosphatase alternative splicing in human breast cancer cell lines. Mol. Oncol..

[15] Ma Y.C., Su N., Shi X.J., Zhao W., Ke Y., Zi X., Zhao N.M., Qin Y.H., Zhao H.W., Liu H.M. (2015). Jaridonin-induced G2/M phase arrest in human esophageal cancer cells is caused by reactive oxygen species-dependent Cdc2-tyr15 phosphorylation via ATM-Chk1/2-Cdc25C pathway. Toxicol. Appl. Pharmacol..

[16] Donzelli M., Draetta G.F. (2003). Regulating mammalian checkpoints through Cdc25 inactivation. EMBO Rep..

[17] Kilpivaara O., Laiho P., Aaltonen L.A., Nevanlinna H. (2003). *CHEK2* 1100delC and colorectal cancer. J. Med. Genet..

[18] Cybulski C., Gorski B., Huzarski T., Masojc B., Mierzejewski M., Debniak T., Teodorczyk U., Byrski T., Gronwald J., Matyjasik J. (2004). *CHEK2* is a multiorgan cancer susceptibility gene. Am. J. Hum. Genet..

[19] Weischer M., Bojesen S.E., Tybjaerg-Hansen A., Axelsson C.K., Nordestgaard B.G. (2007). Increased risk of breast cancer associated with CHEK2*1100delC. J. Clin. Oncol..

[20] Shigeishi H., Yokozaki H., Oue N., Kuniyasu H., Kondo T., Ishikawa T., Yasui W. (2002). Increased expression of CHK2 in human gastric carcinomas harboring p53 mutations. Int. J. Cancer.

[21] Tort F., Hernandez S., Bea S., Martinez A., Esteller M., Herman J.G., Puig X., Camacho E., Sanchez M., Nayach I. (2002). Chk2-decreased protein expression and infrequent genetic alterations mainly occur in aggressive types of non-hodgkin lymphomas. Blood.

[22] Abdel-Fatah T.M., Arora A., Alsubhi N., Agarwal D., Moseley P.M., Perry C., Doherty R., Chan S.Y., Green A.R., Rakha E. (2014). Clinicopathological significance of ATM-Chk2 expression in sporadic breast cancers: A comprehensive analysis in large cohorts. Neoplasia.

[23] Roos W.P., Thomas A.D., Kaina B. (2016). DNA damage and the balance between survival and death in cancer biology. Nat. Rev. Cancer.

[24] Guler G., Himmetoglu C., Jimenez R.E., Geyer S.M., Wang W.P., Costinean S., Pilarski R.T., Morrison C., Suren D., Liu J. (2011). Aberrant expression of DNA damage response proteins is associated with breast cancer subtype and clinical features. Breast Cancer Res. Treat..

[25] Horiuchi D., Kusdra L., Huskey N.E., Chandriani S., Lenburg M.E., Gonzalez-Angulo A.M., Creasman K.J., Bazarov A.V., Smyth J.W., Davis S.E. (2012). MYC pathway activation in triple-negative breast cancer is synthetic lethal with CDK inhibition. J. Exp. Med..

[26] Tarasewicz E., Rivas L., Hamdan R., Dokic D., Parimi V., Bernabe B.P., Thomas A., Shea L.D., Jeruss J.S. (2014). Inhibition of CDK-mediated phosphorylation of Smad3 results in decreased oncogenesis in triple negative breast cancer cells. Cell Cycle.

[27] Liu S., Ginestier C., Charafe-Jauffret E., Foco H., Kleer C.G., Merajver S.D., Dontu G., Wicha M.S. (2008). BRCA1 regulates human mammary stem/progenitor cell fate. Proc. Natl. Acad. Sci. USA.

[28] Deng C.X. (2006). Brca1: Cell cycle checkpoint, genetic instability, DNA damage response and cancer evolution. Nucleic Acids Res..

[29] Carlessi L., Buscemi G., Fontanella E., Delia D. (2010). A protein phosphatase feedback mechanism regulates the basal phosphorylation of Chk2 kinase in the absence of DNA damage. Biochim. Biophys. Acta.

[30] Oka K., Tanaka T., Enoki T., Yoshimura K., Ohshima M., Kubo M., Murakami T., Gondou T., Minami Y., Takemoto Y. (2010). DNA damage signaling is activated during cancer progression in human colorectal carcinoma. Cancer Biol. Ther..

[31] Yajima N., Wada R., Matsuzaki Y., Yagihashi S. (2014). DNA damage response and its clinicopathological relationship in appendiceal tumors. Int. J. Colorectal Dis..

[32] Bartkova J., Guldberg P., Gronbaek K., Koed K., Primdahl H., Moller K., Lukas J., Orntoft T.F., Bartek J. (2004). Aberrations of the Chk2 tumour suppressor in advanced urinary bladder cancer. Oncogene.

[33] Kshirsagar M., Jiang W., Shih Ie M. (2012). DNA damage response is prominent in ovarian high-grade serous carcinomas, especially those with Rsf-1 (HBXAP) overexpression. J. Oncol..

[34] Bartek J., Lukas J. (2003). Chk1 and Chk2 kinases in checkpoint control and cancer. Cancer Cell.

[35] DiTullio R.A., Mochan T.A., Venere M., Bartkova J., Sehested M., Bartek J., Halazonetis T.D. (2002). 53BP1 functions in an ATM-dependent checkpoint pathway that is constitutively activated in human cancer. Nat. Cell Biol..

[36] Poehlmann A., Roessner A. (2010). Importance of DNA damage checkpoints in the pathogenesis of human cancers. Pathol. Res. Pract..

[37] Ke S., Zhou F., Yang H., Wei Y., Gong J., Mei Z., Wu L., Yu H., Zhou Y. (2015). Downregulation of high mobility group box 1 modulates telomere homeostasis and increases the radiosensitivity of human breast cancer cells. Int. J. Oncol..

[38] Farmer H., McCabe N., Lord C.J., Tutt A.N., Johnson D.A., Richardson T.B., Santarosa M., Dillon K.J., Hickson I., Knights C. (2005). Targeting the DNA repair defect in *BRCA* mutant cells as a therapeutic strategy. Nature.

[39] Chiou S.H., Kao C.L., Chen Y.W., Chien C.S., Hung S.C., Lo J.F., Chen Y.J., Ku H.H., Hsu M.T., Wong T.T. (2008). Identification of CD133-positive radioresistant cells in atypical teratoid/rhabdoid tumor. PLoS ONE.

[40] Antoni L., Sodha N., Collins I., Garrett M.D. (2007). Chk2 kinase: Cancer susceptibility and cancer therapy—Two sides of the same coin?. Nat. Rev. Cancer.

[41] Zuo L., Pannell B.K., Re A.T., Best T.M., Wagner P.D. (2015). Po2 cycling protects diaphragm function during reoxygenation via ROS, Akt, ERK, and mitochondrial channels. Am. J. Physiol. Cell. Physiol..

[42] Zuo L., Hallman A.H., Yousif M.K., Chien M.T. (2012). Oxidative stress, respiratory muscle dysfunction, and potential therapeutics in chronic obstructive pulmonary disease. Front. Biol..

